# A decrease in cesarean sections and labor inductions among Swedish women by awareness of fetal movements with the Mindfetalness method

**DOI:** 10.1186/s12884-020-03268-1

**Published:** 2020-10-01

**Authors:** Anna Akselsson, Helena Lindgren, Viktor Skokic, Ingela Rådestad

**Affiliations:** 1 Sophiahemmet University, Stockholm, Sweden; 2 Department of Women and Children’s Health, Karolinska Institutet, Stockholm, Sweden; 3 Institute of Clinical Sciences, Sahlgrenska Academy, University of Gothenburg, Gothenburg, Sweden

## Abstract

**Background:**

Maternal perception of decreased fetal movements is commonly used to assess fetal well-being. However, there are different opinions on whether healthcare professionals should encourage maternal observation of fetal movements, as researchers claim that raising awareness increases unnecessary interventions, without improving perinatal health. We aimed to investigate whether cesarean sections and labor induction increase by raising women’s awareness of fetal movements through Mindfetalness. Further, we aimed to study perinatal health after implementing Mindfetalness in maternity care.

**Methods:**

In a cluster randomized controlled trial, 67 maternity clinics were allocated to Mindfetalness or routine care. In the Mindfetalness group, midwives distributed a leaflet telling the women to focus on the character, strength and frequency of the fetal movements without counting each movement. The instruction was to do so for 15 min daily when the fetus was awake, from gestational week 28 until birth. In this sub-group analysis, we targeted women born in Sweden giving birth from 32 weeks’ gestation. We applied the intention-to-treat principle.

**Results:**

The Mindfetalness group included 13,029 women and the Routine-care group 13,456 women. Women randomized to Mindfetalness had less cesarean sections (18.4% vs. 20.0%, RR 0.92, CI 0.87–0.97) and labor inductions (19.2% vs. 20.3%, RR 0.95, CI 0.90–0.99) compared to the women in the Routine-care group. Less babies were born small for gestational age (8.5% vs. 9.3%, RR 0.91, CI 0.85–0.99) in the Mindfetalness group. Women in the Mindfetalness group contacted healthcare due to decreased fetal movements to a higher extent than women in the Routine care group (7.8% vs. 4.3%, RR 1.79, CI 1.62–1.97). The differences remain after adjustment for potential confounders.

**Conclusions:**

Raising awareness about fetal movements through Mindfetalness decreased the rate of cesarean sections, labor inductions and small-for-gestational age babies.

**Trial registration:**

ClinicalTrials.gov (NCT02865759). Registered 12 August 2016, www.clinicaltrials.gov.

## Background

As a way to prevent stillbirths and other adverse birth outcomes, researchers in the field suggest that pregnant women’s awareness of fetal movements is an important tool [1]. However, Walker and Thornton offer a different opinion, claiming that informing pregnant women about fetal movements is harmful and increases the occurrence of unnecessary interventions [2]. Their critique is based on a randomized controlled trial (AFFIRM) [3], where an intervention that included providing information about fetal movements to the pregnant women, and introducing new guidelines to the clinics, increased the number of labor inductions and cesarean sections. Further, the number of babies in need of neonatal care for more than 48 h was also higher in the intervention group.

In Sweden, midwives are responsible for providing maternal care, and almost all pregnant women attend regularly at no cost. Additionally, if necessary, the women are able to have an extra appointment with an obstetrician at a maternity clinic or, if urgent, at a birth clinic [4]. In the capital, Stockholm, 62% of women who attend maternity care are born in Sweden [5]. These women are at lower risk of negative birth outcomes compared to women born in other countries who have migrated to Sweden [6]. In a cluster-randomized controlled trial, we allocated women to either receive or not receive information about Mindfetalness, a method designed to systematically observe fetal movements. A leaflet including information about fetal movements and instructions on how to practice Mindfetalness were distributed to the women by their midwives. We have previously reported the main analyses for the whole group of women [7]. Our data, as well as our clinical experience, indicate a large number of modifying and possible confounding factors, with many being associated with country of birth. These include knowledge level, cultural attitudes and language comprehension. Here we want to focus on the possible adverse effects of increasing the women’s awareness of fetal movements. To increase validity and the understanding of generalizability, we restricted the analysis to native Swedish-born women. The aim was to investigate whether cesarean section and labor induction increase by raising women’s awareness of fetal movements through Mindfetalness.

## Methods

The study base constitutes pregnant women living in Stockholm, born in Sweden, and who gave birth from 32 weeks’ gestation. The women are drawn from a cluster-randomized controlled trial in which pregnant women, registered at a maternity clinic in Stockholm, Sweden, were randomized to either be informed by their midwife about Mindfetalness or to receive routine care. More information about the method and the randomization process can be found in previous papers [7, 8]. The randomization was carried out via the 78 maternity clinics in the area. Five clinics were excluded because of the small number of women registered annually (< 50) and, additionally, six specialized maternity clinics were excluded. Before the randomization, the maternity clinics were divided into two groups based on the socio-demographics of the area in which the clinics were located; high income area, and non-high-income area. The clinics were further divided based on the number of women registered at each clinic in 2015, the year before recruitment started: small (*n* < 500), medium (*n* = 500–1000) or large (*n* > 1000). Eventually, 33 maternity clinics were randomized to Mindfetalness and 34 maternity clinics to routine care. After the randomization, due to organizational amalgamation, three maternity clinics merged into one clinic and two maternity clinics merged into one clinic, which resulted in a total of 31 maternity clinics in the Routine-care group.

The intervention procedure included the preparation and distribution of a leaflet about fetal movements and the Mindfetalness method to be given to women with singleton pregnancies, registered at a clinic randomized to Mindfetalness (supplementary material). When the leaflets were distributed to the maternity clinics, a lecture about fetal movements and Mindfetalness was delivered to the midwives working at the clinics. A website (www.mindfetalness.com) providing the same information as found in the printed leaflet was made available and open for anyone to access.

The midwives were instructed to hand out the leaflet at a routine visit in gestational week 24 + 0, telling the women to start practicing Mindfetalness from gestational week 28 + 0 and continuing until birth. The women were informed that it was optional for them to use the method and that the quality of maternity care would not be affected by their choice of whether to practice Mindfetalness. Those who wanted to try Mindfetalness were instructed to lie down on their left side for 15 min per day when their unborn baby was awake and to monitor the character, strength and frequency of the fetal movements but not to count each movement. Furthermore, they were asked to trust their intuition and seek care if they felt that the fetal movement pattern had changed, if the movements were weaker, or if the frequency had decreased. All maternity clinics (Mindfetalness clinics and clinics randomized to routine care) continued with standard care, i.e., the midwives provided information (oral not written) about fetal movements in accordance with the guidelines from the Swedish National Board of Health and Welfare [9]; “Healthcare should provide information about fetal movements to all pregnant women in connection with the routine visit to maternal health care offered around 24 weeks’ gestation.” The recommendation is also that “Healthcare should give advice on contacting care again in the event of a renewed experience of decreased fetal movements.” In the leaflet provided to the intervention group, additional information was given, which included, for example, daily systematic observation of fetal movements through the Mindfetalness method, and to focus upon “The intensity of the movements, the way in which the baby moves and how much the baby moves”.

All pregnant women registered at any one of the 33 maternal clinics between 1 November 2016 and 31 January 2018 were included in the analysis. We calculated that, for a training period of at least 4 weeks for the pregnant women using the method, we required a start date at 28 weeks’ gestation and therefore only included women who had given birth from 32 weeks’ gestation in the analysis (thus having completed 4 weeks of Mindfetalness). The observation period was specific to each woman and newborn. We used intention-to-treat analysis. The primary endpoint was labor induction and cesarean section. Secondary endpoints were an Apgar score of zero to six (with stillbirth counting as zero) in the newborn, transfer to neonatal intensive care unit (NICU), small for gestational age, labor from gestation 41 + 6, preterm delivery and “contacting health care due to decreased fetal movements”. The latter was based on the diagnostic coding according to ICD-10 [10] “Examination of decreased fetal movements” (AM041), where no signs of a compromised fetus and no intervention is suggested.

All information was reported to a population-based quality register as part of routine care, regardless of the study [11]. The planning of the study includes two pilot studies [12, 13] and the study is registered with www.ClinicalTrials.gov, number NCT02865759. The study adheres to CONSORT guidelines. Ethics approval was obtained from the The Regional Ethics committee in Stockholm, Sweden (Dnr 2015/2105–31/1).

### Statistical analysis

This is a subgroup analysis of data relating to pregnant women born in Sweden drawn from a large randomized trial [7, 8]. Only register-based data were used. As a metric for association, we calculated percentage ratios. We employed log-binomial regression models to adjust the ratio for possible confounding factors and to calculate 95% confidence intervals. Possible confounding factors comprise age, education level, parity, previous stillbirth, tobacco use at registration, body mass index, assisted reproduction, and maternal diseases. For the analyses, we used statistical program R (version 3.2.4).

## Results

After randomization, one maternity clinic allocated to the intervention declined participation before the study started, but, due to the intention-to-treat design, this maternity clinic was included in the intervention group when analyzing. Approximately 15,500 leaflets were handed out by the midwives during the recruitment period. We received data from 39,865 women with singleton pregnancies who gave birth from gestational week 32 + 0. The Mindfetalness group consisted of a total of 19,639 and, of those, 13,029 were born in Sweden. The corresponding figures for the routine care group were 20,226 and, of those, 13,456 were born in Sweden.

The characteristics for the Mindfetalness group and Routine-care group are displayed in Table 1. The Mindfetalness group included fewer women of advanced age (≥35 years) (27.4% versus 30.6%), and more with previous stillbirths (0.5% versus 0.3%) and tobacco use at registration (3.8% versus 2.9%). The amount of women of normal weight was the same in both groups, but more women had been treated for mental illness in the Mindfetalness group (7.4% versus 5.6%).

**Table 1:** Characteristics of women born in Sweden; 13,029 women registered at a maternity clinic randomized to Mindfetalness and 13,456 women registered at a maternity clinic randomized to routine care

	Mindfetalness *n* (%)	Routine care *n* (%)
Age^a^		
≤ 24	832 (6.4)	694 (5.2)
25–29	3429 (26.3)	3314 (24.6)
30–34	5194 (39.9)	5331 (39.6)
≥ 35	3574 (27.4)	4117 (30.6)
Education level^b^		
Shorter than 9 years	24 (0.2)	17 (0.1)
Elementary school	333 (2.6)	241 (1.8)
High school	3290 (25.3)	3112 (23.1)
University	8784 (67.4)	9090 (67.6)
Parity^c^		
Primipara	5942 (45.6)	6305 (46.9)
Multipara	7021 (53.9)	7105 (52.8)
Previous stillbirth	66 (0.5)	38 (0.3)
Tobacco user at registration at the maternity clinic^c^	500 (3.8)	390 (2.9)
Civic status^c^		
Cohabiting with becoming father	12,211 (93.7)	12,458 (92.6)
Single	141 (1.1)	208 (1.5)
Other family situation	382 (2.9)	467 (3.5)
Body Mass Index^d^		
< 18.5	351 (2.7)	286 (2.1)
18.5–24.9	8289 (63.6)	8481 (63.0)
25.0–29.9	2591 (19.9)	2874 (21.4)
30.0–34.9	855 (6.6)	888 (6.6)
≥ 35.0	354 (2.7)	308 (2.3)
Assisted reproduction^c^	797 (6.1)	889 (6.6)
Maternal diseases		
Diabetes mellitus	17 (0.1)	16 (0.1)
Coronary heart disease	209 (1.6)	229 (1.7)
Thrombosis	110 (0.8)	125 (0.9)
Systemic lupus erythematosus (SLE)	15 (0.1)	29 (0.2)
Psychiatric care	2073 (15.9)	2208 (16.4)
Endocrine disease	845 (6.5)	1007 (7.5)
Epilepsy	62 (0.5)	75 (0.6)
Chronic hypertension	51 (0.4)	79 (0.6)
Other disease	1364 (10.5)	1408 (10.5)
Medication or psychological treatment for mental illness	958 (7.4)	758 (5.6)

^a^Mean: 32.2 vs. 32.6 (SE 0.04 vs. 0.04), Median: 32.0 vs. 32.4, Range 15.7**–**52.0 vs. 16.4**–**52.1, Interquartile range 28.8**–**35.4 vs. 29.3**–**35.8

^b^Missing: Mindfetalness *n* = 598 (4.6%); Routine care *n* = 996 (7.4%)

^c^Missing: Mindfetalness *n* = 66 (0.5%); Routine care *n* = 46 (0.3%)

^d^Missing: Mindfetalness *n* = 589 (4.5%); Routine care *n* = 619 (4.6%)

**Fig. 1:**
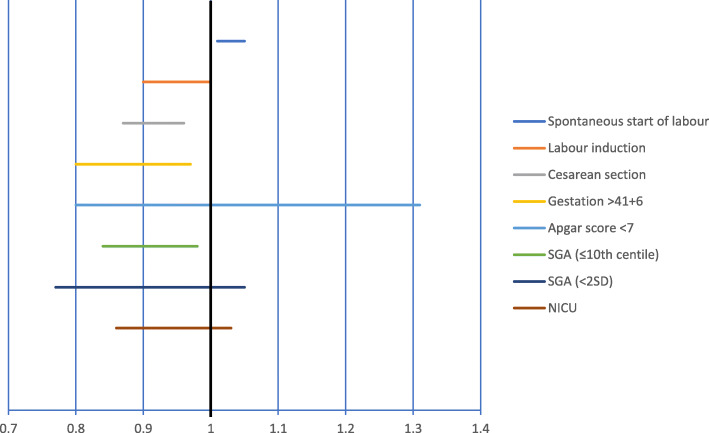
Risk ratio for obstetric outcomes from 32 weeks’ gestation among women born in Sweden randomized to Mindfetalness compared to routine care

As shown in Table 2 and Fig. 1, Swedish women randomized to Mindfetalness had less cesarean sections (18.4% versus 20.0%, RR 0.92, 0.87–0.97) and labor inductions (19.2% versus 20.3%, RR 0.95, CI 0.90–0.99). Further, they started their labor spontaneously to a higher extent (70.9% versus 68.8%, RR 1.03, CI 1.01–1.05). Fewer women in the Mindfetalness group gave birth after 42 weeks’ gestation (5.3% versus 6.0%, RR 0.88, CI 0.80–0.97) and less babies were born small for gestational age (below the 10th centile; 8.5% versus 9.3%, RR 0.91, CI 0.85–0.99). The percentage of babies in need of transfer to neonatal care was lower in the Mindfetalness group, but the difference from the Routine-care group was not statistically significant (6.4% versus 6.8%, RR 0.94, CI 0.86–1.03). Women in the Mindfetalness group contacted healthcare due to decreased fetal movements to a higher extent than women in the Routine care group (7.8% versus 4.3%, RR 1.79, CI 1.62–1.97) (Table 3). The statistically significant differences between the two groups remained after adjustment for age, education, body mass index, previous stillborn child, tobacco use at registration, and treatment for mental illness (calculated one single variable at a time) (Table 3).

**Table 2:** Obstetric outcomes among women born in Sweden with birth from gestation 32 + 0; 13,029 women registered at a maternity clinic randomized to Mindfetalness and 13,456 women registered at a maternity clinic randomized to routine care

Outcome	Mindfetalness *n* (%)	Routine care *n* (%)	Rate Ratio (95% CI)	*p*-value
Induction of labor	2500 (19.2)	2729 (20.3)	0.95 (0.90–0.99)	0.03
Cesarean section (total)	2394 (18.4)	2692 (20.0)	0.92 (0.87–0.97)	< 0.001
Pre-labor	1297 (10.0)	1470 (10.9)	0.91 (0.85–0.98)	0.01
In labor	1097 (8.4)	1222 (9.1)	0.93 (0.86–1.00)	0.06
Spontaneous start of labor	9238 (70.9)	9263 (68.8)	1.03 (1.01–1.05)	< 0.001
Preterm delivery (< 37 + 0)	483 (3.7)	479 (3.6)	1.04 (0.92–1.18)	0.53
Birth gestation > 41 + 6	687 (5.3)	804 (6.0)	0.88 (0.80–0.97)	0.01
Apgar Score < 7 at 5 min^a,d^	127 (1.0)	128 (1.0)	1.02 (0.80–1.31)	0.85
Apgar Score < 4 at 5 min^a,d^	48 (0.4)	39 (0.3)	1.27 (0.83–1.94)	0.28
Birthweight ≤10th centile^b,e^	1107 (8.5)	1253 (9.3)	0.91 (0.85–0.99)	0.02
Birthweight <2SD^c,e^	315 (2.4)	360 (2.7)	0.90 (0.78–1.05)	0.19
Admitted to NICU	832 (6.4)	914 (6.8)	0.94 (0.86–1.03)	0.19
Death within 27 days after birth	2 (0.0)	2 (0.0)	1.02 (0.14–7.26)	1.00

^a^Number of stillbirths: Mindfetalness *n* = 17 (0.1%); Routine care *n* = 14 (0.1%)

^b^International definition of Small for Gestational Age (SGA) ≤10th centile for the gestational age

^c^Swedish definition of Small for Gestational Age (SGA) <2SD from the national reference mean

^d^Missing 46: Mindfetalness 20 (0.2%), Routine care 26 (0.2%)

^e^Missing 23: Mindfetalness 11 (0.1%), Routine care 12 (0.1%)

*NICU* Neonatal intensive care unit

**Table 3:** Obstetric and birth outcomes women born in Sweden with birth from 32 weeks’ gestation; 13,029 women registered at a maternity clinic randomized to Mindfetalness and 13,456 women registered at a maternity clinic randomized to routine care. Obstetric outcomes adjusted for age, educational level, body mass index, previous stillbirth, tobacco use at registration and treatment for mental illness

Outcome	Mindfetalness *n* (%)	Routine care *n* (%)	RR	*p*–value	CI
Induction of labor	2500 (19.2)	2729 (20.3)	0.95	0.03	0.90–0.99
Adjusted for:					
Age			0.95	0.03	0.90–1.00
Educational level			0.93	0.006	0.89–0.98
Body mass index			0.95	0.04	0.90–1.00
Previous stillbirth			0.94	0.02	0.90–0.99
Tobacco use at registration			0.94	0.02	0.90–0.99
Treatment for mental illness			0.94	0.02	0.90–0.99
Cesarean section	2394 (18.4)	2692 (20.0)	0.92	< 0.001	0.87–0.97
Adjusted for:					
Age			0.93	0.006	0.89–0.98
Educational level			0.92	0.002	0.88–0.97
Body mass index			0.92	< 0.001	0.87–0.96
Previous stillbirth			0.92	< 0.001	0.87–0.96
Tobacco use at registration			0.92	< 0.001	0.87–0.96
Treatment for mental illness			0.92	< 0.001	0.87–0.96
Cesarean pre–labor	1297 (10.0)	1470 (10.9)	0.91	0.01	0.85–0.98
Adjusted for:					
Age			0.94	0.06	0.87–1.00
Educational level			0.92	0.02	0.85–0.98
Body mass index			0.91	0.01	0.85–0.98
Previous stillbirth			0.91	0.008	0.85–0.98
Tobacco use at registration			0.91	0.009	0.85–0.98
Treatment for mental illness			0.91	0.007	0.85–0.97
Cesarean in labor	1097 (8.4)	1222 (9.1)	0.93	0.06	0.86–1.00
Adjusted for:					
Age			0.93	0.07	0.86–1.01
Educational level			0.93	0.08	0.86–1.01
Body mass index			0.92	0.04	0.85–1.00
Previous stillbirth			0.93	0.05	0.86–1.00
Tobacco use at registration			0.92	0.05	0.85–1.00
Treatment for mental illness			0.92	0.05	0.85–1.00
Spontaneous start of labor	9238 (70.9)	9263 (68.8)	1.03	< 0.001	1.01–1.05
Adjusted for:					
Age			1.02	0.002	1.01–1.04
Educational level			1.03	< 0.001	1.02–1.05
Body mass index			1.03	< 0.001	1.01–1.04
Previous stillbirth			1.03	< 0.001	1.02–1.05
Tobbacco use at registration			1.03	< 0.001	1.01–1.05
Treatment for mental illness			1.03	< 0.001	1.01–1.05
Preterm delivery	483 (3.7)	479 (3.6)	1.04	0.52	0.92–1.18
Adjusted for:					
Age at birth			1.04	0.55	0.92–1.18
Educational level			1.04	0.55	0.91–1.18
Body mass index			1.05	0.44	0.93–1.20
Previous stillbirth			1.04	0.54	0.92–1.18
Tobacco user at registration			1.04	0.55	0.92–1.18
Treatment for Mental illness			1.04	0.55	0.92–1.18
Gestation > 41 + 6	687 (5.3)	804 (6.0)	0.88	0.01	0.80–0.97
Adjusted for:					
Age at birth			0.88	0.01	0.80–0.97
Educational level			0.88	0.02	0.80–0.98
Body mass index			0.88	0.02	0.80–0.98
Previous stillbirth			NA	NA	NA
Tobacco user at registration			0.88	0.01	0.80–0.97
Treatment for Mental illness			0.89	0.02	0.81–0.98
Apgar score less than 7 at 5 min^a^	127 (1.0)	128 (1.0)	1.02	0.85	0.80–1.31
Adjusted for:					
Age at birth			1.02	0.88	0.80–1.30
Educational level			1.01	0.93	0.78–1.30
Body mass index			0.99	0.96	0.77–1.27
Previous stillbirth			1.02	0.86	0.80–1.31
Tobacco user at registration			1.02	0.86	0.80–1.31
Treatment for Mental illness			1.00	0.99	0.78–1.28
Apgar score less than 4 at 5 min^a^	48 (0.4)	39 (0.3)	1.27	0.26	0.83–1.95
Adjusted for:					
Age at birth			1.27	0.27	0.83–1.94
Educational level			1.26	0.30	0.81–1.98
Body mass index			1.31	0.21	0.86–2.01
Previous stillbirth			1.27	0.27	0.83–1.94
Tobacco user at registration			1.27	0.26	0.84–1.95
Treatment for Mental illness			1.25	0.29	0.82–1.92
Birthweight ≤10th centile	1107 (8.5)	1253 (9.3)	0.91	0.02	0.84–0.98
Adjusted for:					
Age at birth			0.91	0.01	0.84–0.98
Educational level			0.90	0.01	0.83–0.98
Body mass index			0.90	0.01	0.84–0.98
Previous stillbirth			0.91	0.02	0.84–0.98
Tobacco user at registration			0.91	0.01	0.84–0.98
Treatment for Mental illness			0.91	0.02	0.84–0.98
Birthweight <2SD^b***^	315 (2.4)	360 (2.7)	0.90	0.18	0.78–1.05
Adjusted for:					
Age at birth			0.90	0.16	0.77–1.04
Educational level			0.91	0.26	0.78–1.07
Body mass index			0.89	0.14	0.77–1.04
Previous stillbirth			0.90	0.19	0.78–1.05
Tobacco user at registration			0.90	0.17	0.77–1.04
Treatment for Mental illness			0.90	0.19	0.78–1.05
Transfer to NICU	832 (6.4)	914 (6.8)	0.94	0.18	0.86–1.03
Adjusted for:					
Age at birth			0.94	0.19	0.86–1.03
Educational level			0.95	0.24	0.86–1.04
Body mass index			0.95	0.28	0.87–1.04
Previous stillbirth			0.94	0.18	0.86–1.03
Tobacco user at registration			0.94	0.17	0.86–1.03
Treatment for Mental illness			0.93	0.15	0.85–1.02
Death within 27 days after birth	2 (0.0)	2 (0.0)	1.03	0.97	0.12–8.61
Adjusted for:					
Age at birth			1.07	0.94	0.13–8.96
Educational level			1.01	0.99	0.12–8.43
Body mass index			1.07	0.95	0.13–8.88
Previous stillbirth			NA	NA	NA
Tobacco user at registration			NA	NA	NA
Treatment for Mental illness			NA	NA	NA
Contacting healthcare due to DFM	1011 (7.8)	584 (4.3)	1.79	< 0.001	1.62–1.97
Adjusted for:					
Age at birth			1.77	< 0.001	1.60–1.95
Educational level			1.83	< 0.001	1.65–2.03
Body mass index			1.81	< 0.001	1.63–2.00
Previous stillbirth			1.79	< 0.001	1.62–1.98
Tobacco user at registration			1.79	< 0.001	1.62–1.97
Treatment for Mental illness			1.79	< 0.001	1.62–1.98

^a^Data are missing for 46 women (20 in Mindfetalness group and 26 in Routine care group)

^b^Data are missing for 23 women (11 in Mindfetalness group and 12 in Routine care group)

*Number of stillbirths (Apgar = 0) Mindfetalness *n* = 17 (0. 1%); Routine care *n* = 14 (0. 1%)

**International definition of Small for Gestational Age (SGA) ≤10th centile for the gestational age

***Swedish definition of Small for Gestational Age (SGA) <2SD from the national reference mean

*NICU* Neonatal intensive care unit

## Discussion

This analysis of Swedish-born women in a population-based prospective trial using cluster randomization showed no indications of harm when midwives encouraged awareness of fetal movements. On the contrary, by raising awareness through Mindfetalness, labor induction and cesarean sections decreased and, further, the number of women having a small-for-gestational-age baby also decreased.

Reflecting the debate in Scandinavia, an associate editor of *Acta Obstetricia et Gynecologica Scandinavica* recently promoted a strategy in Sweden of not encouraging women to pay attention to their unborn baby’s movements [14]. To better understand the possible effects of such a strategy, we restricted the analysis to Swedish-born women, avoiding possible confounding or modifying effects by including women not born in Sweden. We found no indication that being aware of fetal movements causes harm. We believe that the debate that emerged after the AFFIRM-trial has distorted the concept of maternal awareness. The trial had two interventions presented as one; one aimed towards health-care professionals and one towards women, of increasing awareness of fetal movements [3]. These interventions were performed at the same time. Moreover, although not statistically significant, it is important to note that the two interventions together resulted in a stillbirth rate of 4.06 per thousand births, compared to 4.40 in standard care. Further, concerning the AFFIRM trial, it has been argued that almost 40% of the maternity clinics had low compliance to the intervention guidelines. Our study comprised one intervention only; midwives in half of the maternity clinics promoted Mindfetalness as a means of increasing awareness of fetal movements. We found a decreased rate of caesarean section and labor induction. We have no indication that clinical practice in managing women with decreased fetal movements was any different in the two groups we compared. The obstetricians did not know which maternity clinics were randomized to our intervention with Mindfetalness. We believe the way forward differs to what Walker and Thornton [2] and Gidlöf [14] suggest. Taken together, the results of the AFFIRM study, our study, and the one conducted by Tveit and co-workers [15], do not support concerns that increased awareness of fetal movements causes harm. Additionally, in a recent systematic review and meta-analysis investigating the effect of fetal movement counting in perinatal mortality and obstetric outcomes, an 8 % reduction in perinatal mortality was seen in the fetal movement counting group (CI 0.85–1.00) [16]. The rate of labor induction and cesarean section was somewhat higher in the counting group. The participants of one study contributed to 82% of the total number of participants across all studies included in the review, which the authors regarded as a limitation. A comparison of the Mindfetalness-trial with the results of this review is difficult, as our study design and the concept “awareness of fetal movements” differs from those of the studies included in the article by Belussi et al. [16].

We found that less women had small-for-gestational-age infants in the Mindfetalness group. One explanation for this observation might be correlated to the instructions for practicing Mindfetalness. In the leaflet, the women were instructed to observe fetal movements on a daily basis, lying down on their side, to optimize blood flow to the placenta. Women who read the leaflet might have therefore adopted this position when going to sleep, rather than a supine position. Researchers have found an association between supine sleep position and reduced birthweight and even stillbirth [17, 18].

### Strengths and limitations

Our effect measures are somewhat diluted because not all of the women randomly allocated to Mindfetalness practiced it. From the knowledge obtained from the original Mindfetalness trial and its pilot study [12], we know that approximately 78% of the leaflets were distributed (all languages included) and about 75% of the women practiced the method. Moreover, possible contamination between the two groups studied is an issue. Pregnant women change maternity clinics during pregnancy, and midwives change their workplace. Additionally, the website was open for anyone to access, and media highlighted Mindfetalness during the study period. Further, participating in research about fetal movements may have had a potential effect on the midwives’ behavior, both in the intervention and the routine care arm [19, 20]. This type of misclassification may have diluted the results further, and the measured differences will in fact be greater in reality. The differences between the groups remain statistically significant, even after adjustment for confounders, and this strengthens the results, although is it possible that we have not identified all factors that could have affected the results. In moving forward, we may need to find essential components of the methods used to promote awareness of fetal movements. That may give us an understanding of the discrepancies between the varying results in different published studies. Perhaps the beneficial effects we have had are due to the fact that Mindfetalness (focusing on the strength, character and frequency of the movements but not count each movement) was a helpful tool for the women to get to know their unborn baby’s movement pattern and to trust their intuition. We believe all evidence we have support that when women are aware of fetal movement, pregnancy outcome improves.

## Conclusion

Our intervention increased the percentage of women seeking obstetric care for decreased fetal movements. If women with decreased fetal movements are more likely to seek care earlier, new opportunities emerge. Our data clearly showed no harm by increasing maternal awareness of fetal movements, on the contrary, we saw benefits. We may still have a lot to learn about how to help a woman improve her knowledge about her unborn baby’s movement pattern and how a fetal-movement history can be translated to an even better pregnancy outcome than exists today.

## Supplementary information

**Additional file 1.**

## Data Availability

The Ethics committee prohibit data to be publicly available due to confidential information. However, the data will be shared after an approval from the Regional Ethics committee in Stockholm, Sweden (https://etikprovningsmyndigheten.se/).

## Abbreviations

NICU: Neonatal intensive care unit
SGA: Small for gestational age
